# Visual and Physical Degradation of the Black and White Mosaic of a *Roman Domus* under Palazzo Valentini in Rome: A Preliminary Study

**DOI:** 10.3390/molecules27227765

**Published:** 2022-11-11

**Authors:** Claudia Colantonio, Paola Baldassarri, Pasquale Avino, Maria Luisa Astolfi, Giovanni Visco

**Affiliations:** 1 Department of Chemistry, University of Rome “La Sapienza”, p.le Aldo Moro 5, I-00185 Rome, Italy; 2 Città Metropolitana di Roma Capitale, U.C. 2, Servizio 3, Palazzo Valentini, Via Quattro Novembre 119a, I-00187 Rome, Italy; 3 Department of Agricultural, Environmental and Food Sciences (DiAAA), University of Molise, Via De Sanctis, I-86100 Campobasso, Italy; 4 Research Center for Applied Sciences to the safeguard of Environment and Cultural Heritage (CIABC), University of Rome “La Sapienza”, p.le Aldo Moro 5, I-00185 Rome, Italy

**Keywords:** *Roman domus*, mosaic restoration, multi-method diagnostic, NMR, FT-IR, GC-MS

## Abstract

Palazzo Valentini, the institutional head office of Città Metropolitana di Roma Capitale, stands in in a crucial position in the Roman archaeological and urban contexts, exactly between the Fora valley, Quirinal Hill slopes, and Campus Martius. It stands on a second-century A.D. complex to which belong, between other archeological remains, two richly decorated aristocratic domus. One of these buildings, the *domus A*, presents an outward porticoed room with a fourth-century AD central *impluvium* (open air part of the atrium designed to carry away rainwater) with a black/white tiled mosaic pavement, the preservation status of which is compromised by an incoherent degradation product that has caused gradual detachment of the mosaic tiles. To identify the product and determine the causes of degradation, samples of the product were taken and subjected to SEM-EDS, XRF, NMR, FT-IR and GC-MS analyses. The findings reported in this study can help restorers, archaeologists and conservation scientists in order to improve knowledge about the Roman mosaic, its construction phases, conservation problems and proper solutions.

## 1. Introduction

Roman mosaic is one of the most beautiful expressions of ancient art that has survived to this day. The etymology of the word “mosaic” comes from “Muse”, which was related to the parietal decorations of the caves consecrated to the Muses that were built in Roman gardens. The art of mosaic [1], as a decorative technique, found its first expression in the Aegean culture in productions of natural colored pebble pavements, initially grounded without stone processing and later carved to realize more refined geometries.

The retaining structure (*substructio*) for a mosaic pavement installation was made up of three layers: a primer coat called *statuminatio*, consisting of a conglomerate of big pieces of rocks with a fist-like size; a second layer, almost 25-cm deep, called *rudus*, made up of three parts of gravel and one part of lime; a nucleus, almost half the depth of the previous layer, composed of three parts of hydraulic lime mixed to crushed pottery and one part of lime. This use of crushed pottery together with lime was defined *opus signinum* by Romans, so-called from the town of Signia, renowned for its earthenware (“terracotta”) shingles, and represented an innovation, compared with Greek mosaic pavements cemented with lime, thanks to which mosaic basements gained major resistance and stability.

Tiles were inserted into a superficial plaster layer and their surface was made perfectly smooth using appropriate tools to level them off. The last step involved applying a mixture of marble powder, sand and lime to produce a compact and tough surface.

Some beautiful examples of late Roman mosaic art, belonging to the floors of two residential rooms, were found in 2005 in the underground excavations of Palazzo Valentini in Rome [2,3,4], together with a private thermal complex, both pertaining to the fourth-century A.D. phase, and most significantly offering two rich aristocratic domus.

**Figure 1:**
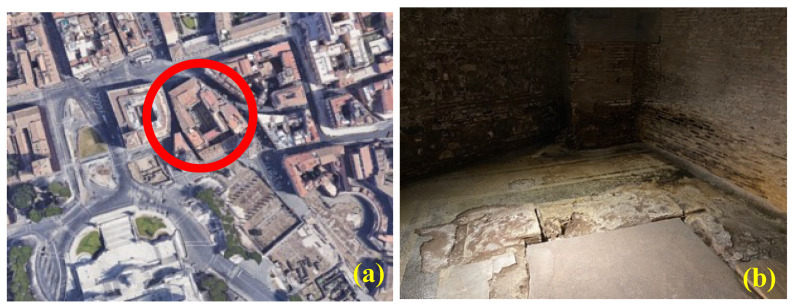
In the red circle is evidenced Palazzo Valentini (source: Google Earth [9]) (**a**). The paviment mosaic and the immediately facing wall (**b**).

Palazzo Valentini (Figure 1a), rising at a crucial point regarding Roman archaeological and urban contexts, was found exactly between the Fora valley, Quirinal Hill slopes and Campus Martius, and was built by Cardinal Michele Bonelli, starting from 1585, over a preexisting building, Palazzo Zambeccari, from the middle of the sixteenthth century, that he bought from Giacomo Boncompagni [5,6,7]. The great enlargement and renovation work commissioned by Cardinal Bonelli were within the perspective of the supremacy of a nerve center in the town [8], as confirmed by the Roman cartography of the time, i.e., Antonio Tempesta’s “*Veduta di Roma*”, 1593 (Figure S1 of the Supplementary Material). In 1827, the palace was bought by a banker, Vincenzo Valentini, to be his house, fostering the conclusion of works towards the Fora.

The most significant and fascinating findings were the rooms pertaining to at least two buildings of the middle and late Imperial Age, which were surely configurated as *domus A* and *domus B* in the fourth century A.D.: indeed, their construction must be dated between the reigns of Hadrian and Septimius Severus (117–211 A.D.), but their story continues until the end of the fifth century A.D. [10].

The prestige of these two residences in the first half of the fourth century A.D. is immediately confirmed by the richness of decorative setup, consisting of great mosaic pavements in the *peristilium* (Figure S2a of the Supplementary Material) and the *triclinium* (Figure S2b of the Supplementary Material) of *domus A*, and in the marble covering of a staircase and an apsidal Aula in the *domus B*. The walls and pavements were covered by refined opus sectile with different polychrome marbles, which provided the room with the aspect of a boardroom.

**Figure 2:**
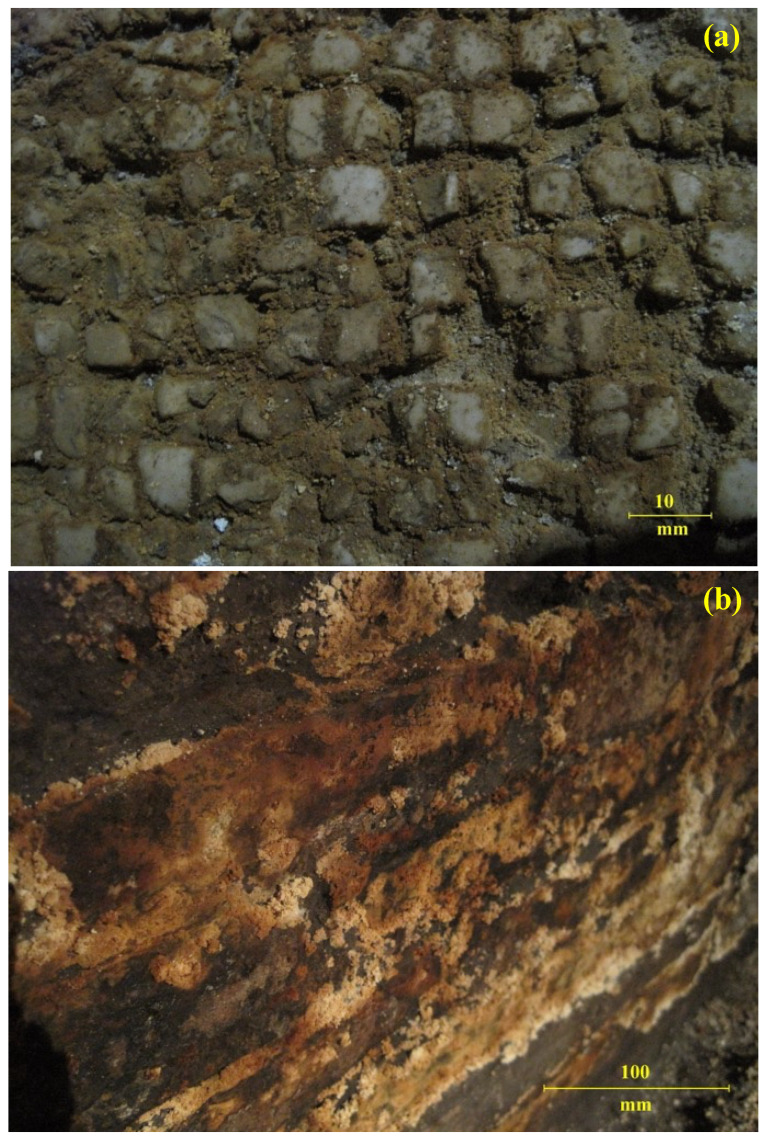

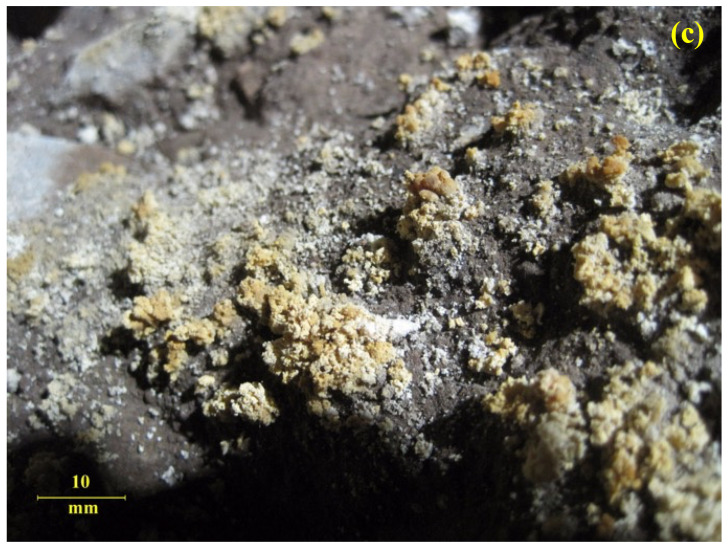
Photos of the degradation (**a**) of the floor and (**b**) of the wall and optical microscope image (**c**) on the surface of the black and white mosaic pavement (10× and 40× magnification, respectively; width focusing 25 mm, 2 mm increments; 6 V/20 W halogen light). The sample of the degradation product of the black and white pavement mosaic of *domus B* was analyzed under an optical microscope in transmittance and reflectance).

The mosaic room is located at a depth of about 5.5 m under the modern street level and is enclosed on three sides by two walls dating to about 1865, and one perimetral wall of the palace, which has a basement window at its upper end, opening onto the road. The state of decay is clearly visible in the photographs and is not present in any other area of the excavation (Figure 2).

Although the excavation areas investigated up to this day are still limited, compared with the total extension of the area of interest, the quality and quantity of archaeological remains allow us to assert with confidence that they belonged to an important residential quarter of senators or dignitaries of the imperial court, which occupied the most part of the NW and NE areas of the Trajan’s Forum.

The paper focuses on the state of chemical degradation of the mosaic’s right part and the part immediately facing the wall (Figure 1b–d). The need to identify the causes of chemical degradation is important for the historical reconstruction of the use of this environment over time and for the correct restoration of the wall and, essentially, the mosaic itself. This paper shows the preliminary results in the possible identification of the compounds responsible for such degradation.

Further, it would be advantageous that all the operations were coordinated among restorers, archaeologists, and conservation scientists in order to improve our knowledge about the Roman mosaic, its construction phases, conservation problems, and proper solutions to the problems.

Finally, it should be underlined that the archaeological excavation campaign started eight years ago and has assumed unexpected dimensions and the perfect state of conservation of the structures has made it possible to undertake work to enhance the site and open it to the public with the offer of a sophisticated multimedia system as virtual support to the museum itinerary.

## 2. Results

During a study led by one of the authors to contrast micro-biological and micro-flora growth, typical of hypogeum settings, by different enlightenment systems (the authors apologize but the procedure and data cannot be displayed because they are under international patent process/review, although some information can be found at ref. [11]), the widespread presence of brown spots was observed and ascribed to some biodegradation products but later assessed differently. As a result, the geometric black and white mosaic of *domus A* was completely restored in 2007 (though without replenishing the detached tiles, still present on the surface) to allow a clear reading for visitors.

By accurate examination, it was possible to observe that the mosaic tiles were intact, and the degradation product was lying over the surface of the tile and in the interstices between one tile and another; this suggested that the degradation mainly concerned the mosaic mortar layer, essentially made up of calcium carbonate (CaCO_3_).

Degradation also extended to the room walls (Figure 2a,b) and seemed similar to that affecting the pavement. Over the centuries (especially after the sixth century), the functional use of these underground settings, located at between 3.5 and 5.5 m of depth in respect to the current ground level, is unclear because of fragmented or missing documentation.

**Figure 3:**
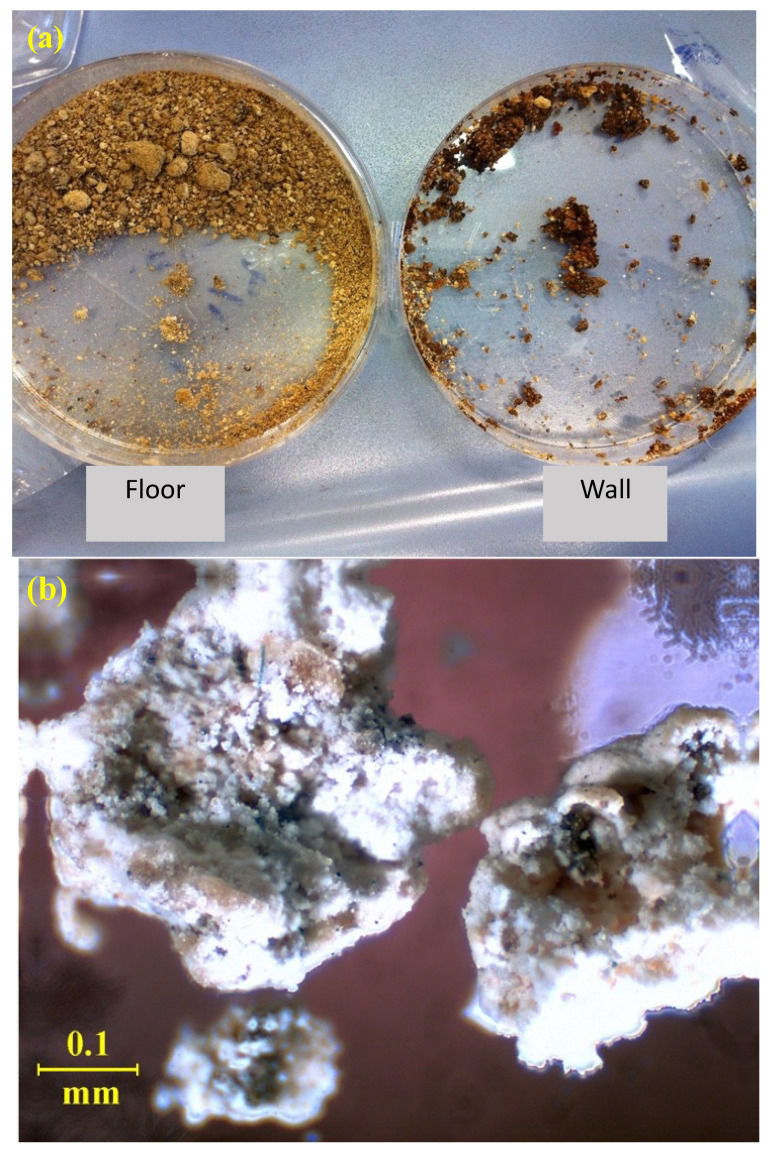
(**a**) Photo of the samples of the degradation product of both the floor and the wall (glass Petri plate 80 mm); (**b**) photo of the degradation product of the pavimental mosaic taken by the optical microscope (1000× magnification; width focusing 25 mm, 2 mm increments; 6 V/20 W halogen light).

By OM, it has been possible to highlight a substantial homogeneity of the degradation product, consisting of small grain aggregate with porous surface (Figure 3). Nevertheless, the mosaic tiles are entirely covered by a yellowish-brown degradation product of incoherent substance (Figure 2a,c), more damp and compact at some pavement points. In addition to the visual and aesthetic damage, there is the implication of a loss of mechanical–structural properties and, therefore, of binding power.

**Figure 4:**
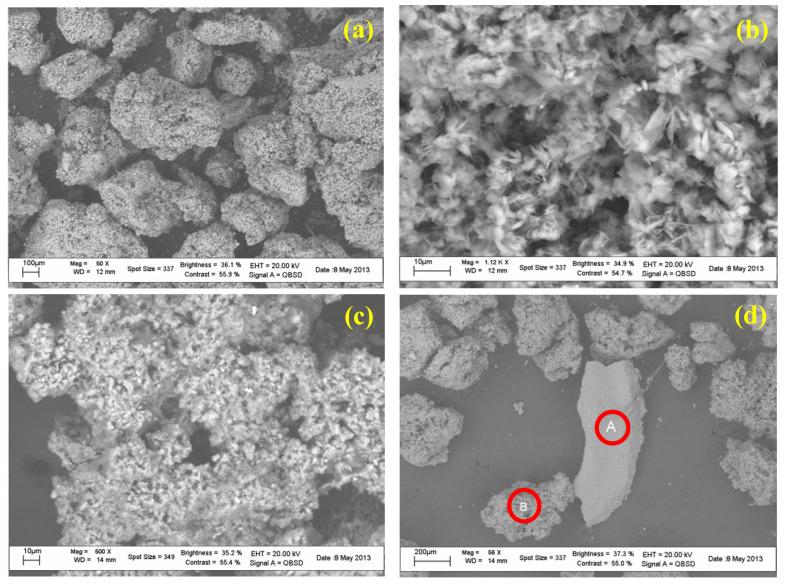
SEM micrographs of the samples collected from pavimental mosaic (**a**,**b**) and from the wall (**c**,**d**).

The samples taken from the pavimental mosaic and the wall were observed by SEM at magnifications from 50× up to over 1000× to highlight the crystalline structure. The SEM analysis confirmed the porous structure observed by means of optical microscope. EDS microprobe analyses were then used to identify the elemental composition of the samples. Figure 4a,b show the SEM images of the sample from the pavimental mosaic, whereas Figure 4c,d show the SEM images of the sample from the wall. Table 1 reports the EDS results obtained for both the floor and wall samples.

The difference between gypsum morphology and CaSO_4_ and CaCO_3_ morphologies are important issues important at this point, requiring some consideration. Basically, it should be remembered that the relationship between the thermodynamic concentrations of Ca for pure CaCO_3_ and CaSO_4_ solutions is dependent on the pH of the CaCO_3_ solution. CaSO_4_ precipitated in the form of gypsum has a needle shaped structure, while CaCO_3_ has a spiral growth and precipitates in the form of calcite. The precipitate structure is affected by the co-existence of salts. Their co-precipitation results in CaCO_3_ crystals interwoven with CaSO_4_ crystals. This tends to result in a co-precipitate that is stronger than pure CaSO_4_ and weaker than pure CaCO_3_ precipitate [12]. EDS spectra showed that the sample from the pavement was almost entirely made up of gypsum (calcium sulphate, CaSO_4_) with well grown and homogeneous crystals of small dimensions. A small quantity of calcium carbonate (CaCO_3_) was also revealed, the occurrence of which can be ascribed to the mosaic mortar, since the tile surfaces and bodies look intact, although moved along from their inclusion points in the mosaic pavement. On the basis of such observation, it can be supposed that the tiles have slipped from their location because of pressure exerted by the degradation product (of a chemical or biological nature), which caused a physical degradation with structural failure of the mortar [13,14].

**Figure 5:**
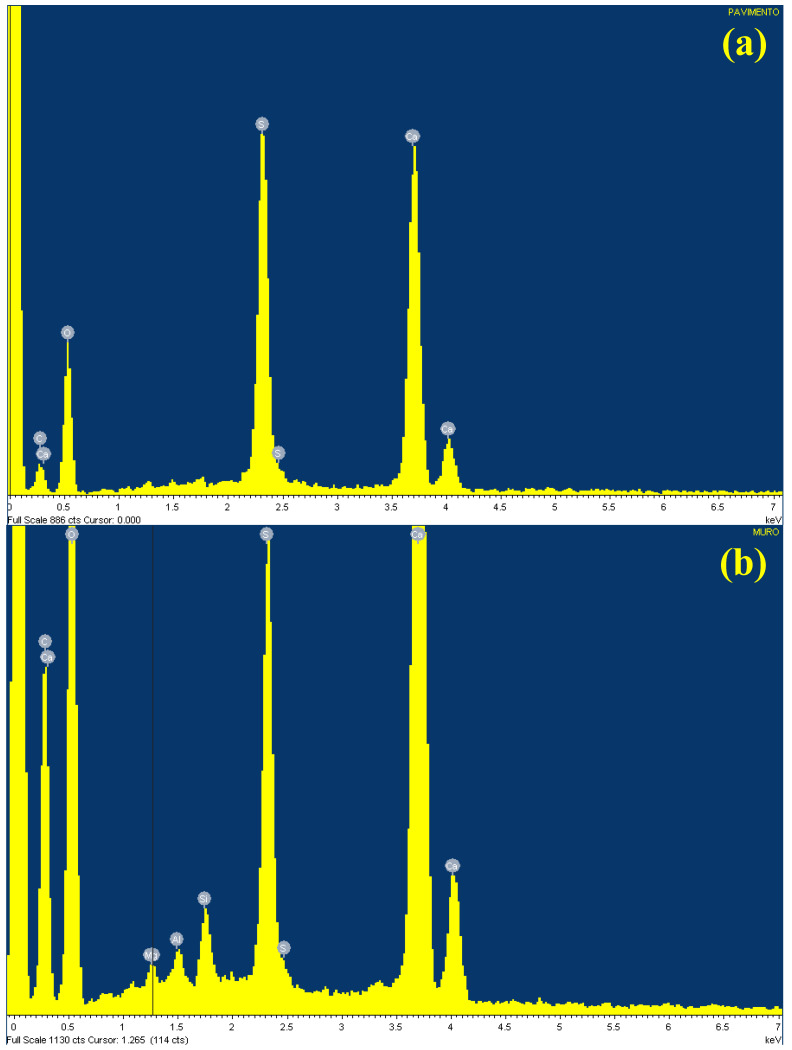
EDS spectra of (**a**) floor sample and (**b**) wall sample. For experimental condition: see Section 4 (Materials and Methods).

The sample from the wall (Figure 4c,d) looks similar to the pavement one but contains some fragments of a more compact aspect (zone A in Figure 4d). EDS spectra (Figure 5a,b, Table 1) revealed a lower gypsum content and a higher one of calcium carbonate. There is also a component, albeit minimal, of Mg (0.3%), Al (0.3%), and Si (0.8%), feldspars probably imputable to grains of earth.

**Figure 6:**
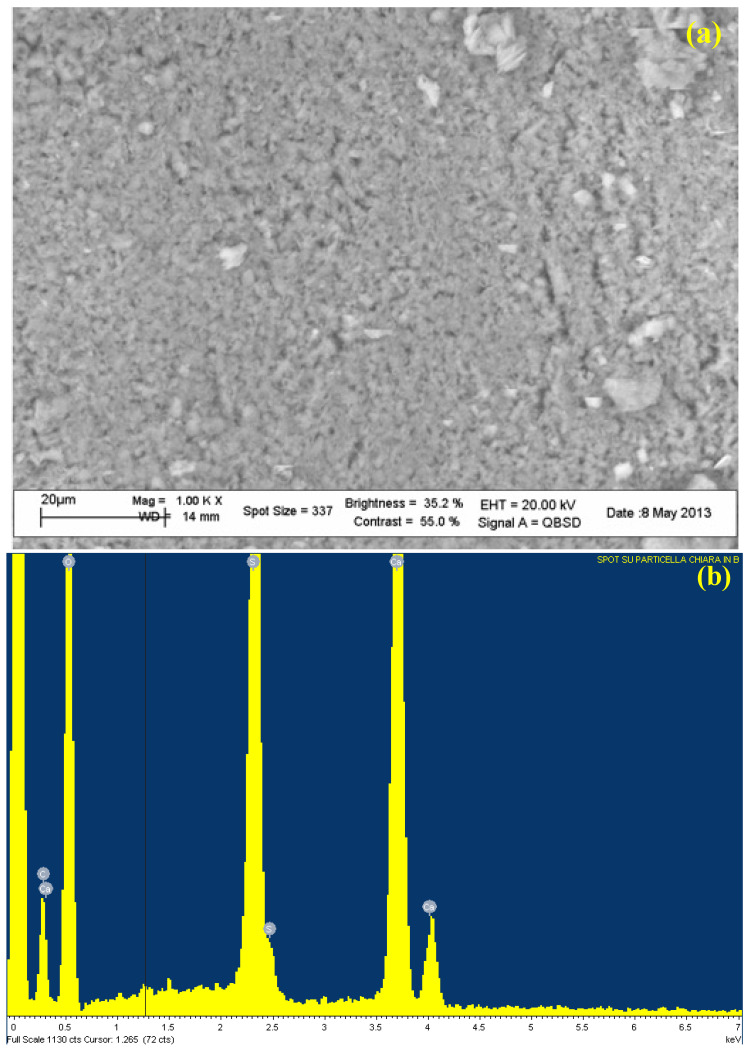
SEM photo (**a**) and EDS spectrum (**b**) of the spot of the particle “A” of the wall sample. For experimental condition: see Section 4 (Materials and Methods).

The more compact portion (particle “A”) differs from the others (particle “B”), with a higher gypsum content and higher calcium carbonate (Figure 6b and Table 1). At higher magnification (1000×) (Figure 6a), as expected very small grains became visible. Another significant difference between the pavement and wall samples was the higher content of Carbon, which cannot be fully imputed to the higher carbonate content because of the lower Calcium (which, as for the pavement sample also cannot satisfy both gypsum and carbonate stoichiometry), so revealing a higher content of organic compounds (Table 1).

**Figure 7:**
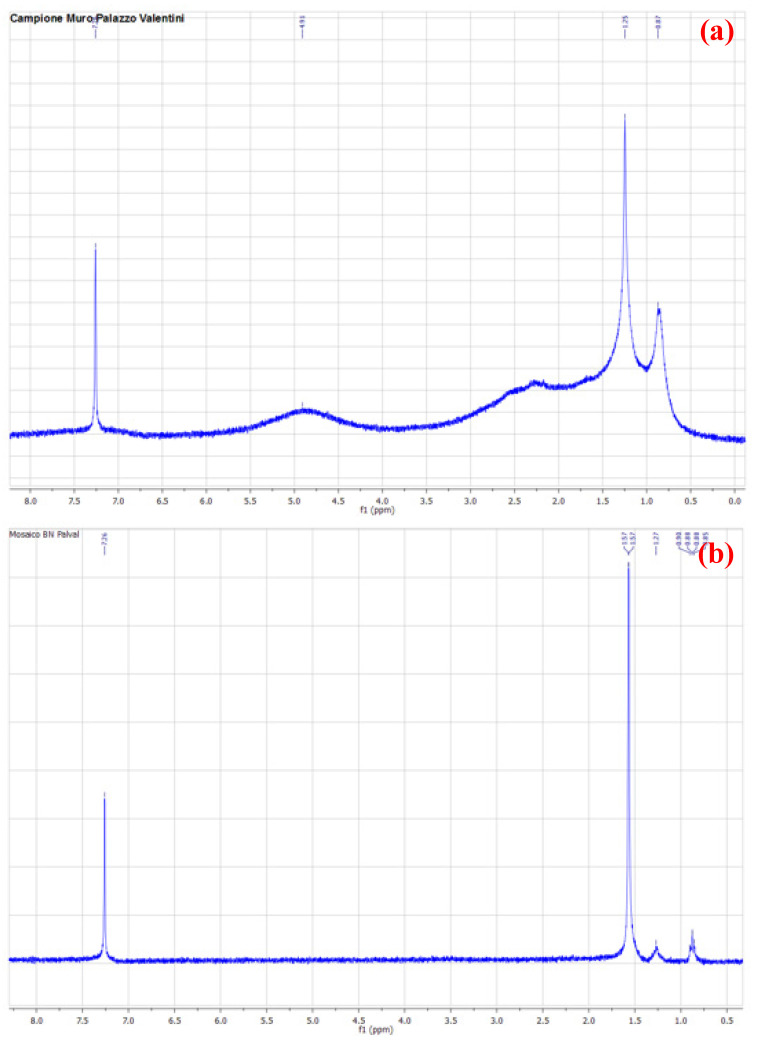
^1^H-NMR spectra (300 MHz, CDCl_3_) of (**a**) wall and (**b**) pavement samples.

Indeed, the yellowish-brown color of the degradation product, and the possible use of the room as a deposit for materials, whose composition or consequences of their use or abandonment were not clear, strengthens the presence of hydrocarbon compounds in the samples collected from the pavimental mosaic and the wall. Following this hypothesis, NMR analyses of pavement and wall samples were performed to identify contingent organic compounds (Figure 7a,b) [15].

Analysis of the NMR spectra showed the presence of aliphatic hydrocarbons in the samples (shift at 7.3 ppm is deuterated chloroform used as solvent). In particular, Figure 7b clearly shows the presence of aliphatic hydrocarbons (shifts between 0.8 and 1.7 ppm). On the other hand, Figure 7a shows a broad signal. The presence of some -OH group caused a shift broadening. Basically, protons on carbon adjacent to the alcohol oxygen showed up in the region of 3.4–4.5 ppm. The electronegativity of the alcohol oxygen de-shielded these protons causing them to appear downfield, when compared to alkane protons. Further, protons directly attached to the alcohol oxygen often appeared in the region of 2.0 to 2.5 ppm. These peaks tended to appear as short, broad singlets [16,17]. Actually, there was also another reasonable interpretation of these broad signals in the NMR spectrum:. They may also be the product of a larger molecular size of the compound or the presence of some paramagnetic impurities in the sample. In any case, the identification of the substance was carried out through a comparison with the online database of ^1^H-NMR spectra of organic substances in CDCl_3_ provided by the National Institute of Advanced Industrial Science and Technology (AIST). From the analysis of the ^1^H-NMR spectra, the presence of aliphatic hydrocarbons seemed to emerge, some of which also contained amino and sulfur groups, such as octane, 2-methylhexane, 2-mthylnonane, decylamine, 1-methyleptylamine, methylpentylsulfide, sodium 1-octanesulfonate (Figure S3 of the Supplementary Material), and, mainly, a more complex composition in the sample coming from the wall (Figure 7a) than that coming from the pavement (Figure 7b).

**Figure 8:**
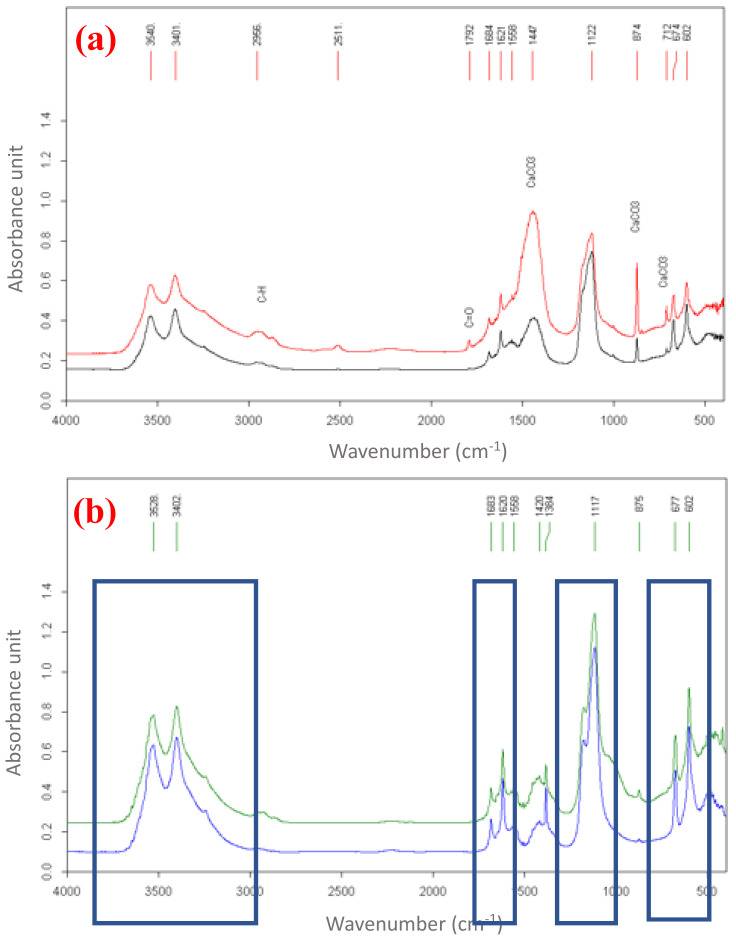
FT-IR spectra (4000–400 cm^−1^; Globar source and deuterated triglycine sulfate, DTGS, detector; 200 scans; resolution 2 cm^−1^) of samples taken from (**a**) wall and (**b**) floor (CaCO_3_ 875 cm^−1^; CaSO_4_ 607 cm^−1^). The red/black and green/blue spectra in figure (**a**) and figure (**b**), respectively, are related to two different samples from wall (**a**) and floor (**b**). The blue boxes show the signals due to calcium sulfate.

The FT-IR analyses were conducted by putting the samples from the wall (Figure 8a) and floor (Figure 8b) on potassium bromide (KBr) tablets. The spectra (recorded in the range 4000–400 cm^−1^) confirmed the presence of calcium sulfate (by diagnostic peaks at 607, 1117, 1620 cm^−1^) in the product lying on the mosaic pavement (Figure 8b). The calcium carbonate peak (at 875 and 1384 cm^−1^) was attributed to mosaic mortar, while the double peak at 3400–3500 cm^−1^ was due to a N–H simple bond in the compound.

The FT-IR analysis of the samples coming from the wall (Figure 8a) and floor (Figure 8b) showed that their hydrocarbon compositions were essentially similar. The possible reason for this was common contamination. In addition, the study of the FT-IR spectra also indicated that the sample coming from the floor contained only gypsum, whereas that coming from the wall also contained calcium carbonate, which was associated with the binder of the mortar that constituted it. Further, the FT-IR spectra also demonstrated the presence of possible compounds containing N–H: these compounds could be the same as those identified by NMR analysis and reported in the Supplementary Material, namely 1-methylheptylamine and decylamine.

**Figure 9:**
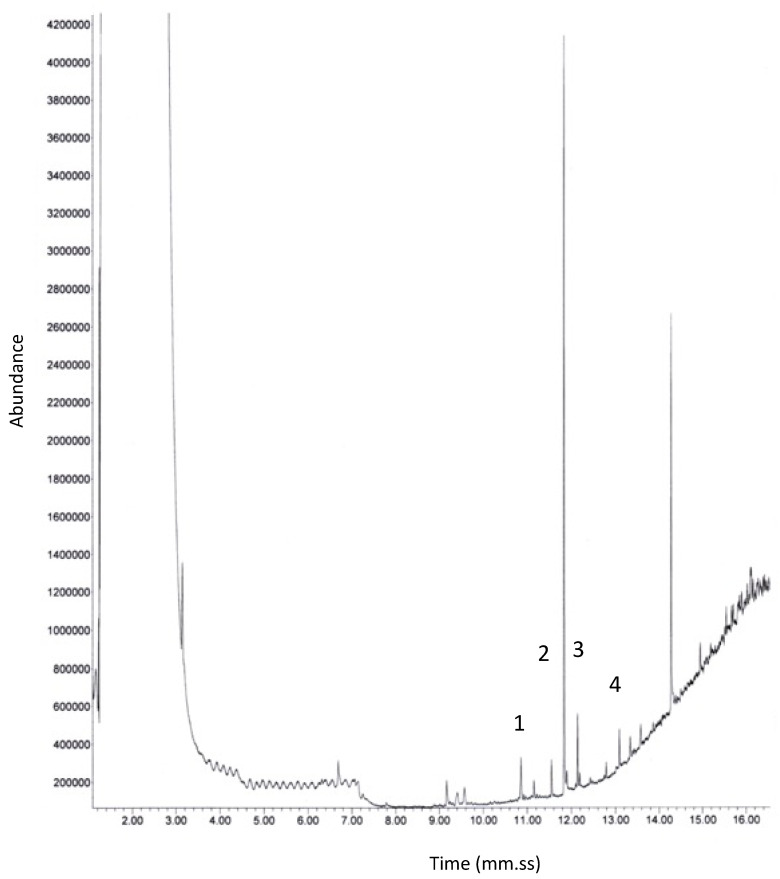
GC-MS chromatogram (fused-silica capillary column, HP-5MS; stationary phase SE54; 30 m × 0.25 mm I.D. and 0.25 µm d_f_; T_inj_ 300 °C; splitless mode 30 s; T transfer line 200 °C; program: T_0_ 120 °C, isothermal for 1 min, 30 °C min^−1^ up to 200 °C, 5 °C min^−1^ up 230 °C, then 30 °C min^−1^ up to T final 290 °C; carrier gas He; *m/z* from 50 to 700 at 70 eV.) of a pavement sample. Peaks: 1 hexadecane, 2 octadecane, 3 nonyl 2-propyl ester sulfurous acid, 4 tributylchloro-stannane. Mass spectra are shown in Figure S4 of the Supplementary Material.

Finally, the GC-MS analysis on a floor sample subjected to chemical degradation made it possible to identify the organic composition responsible for the yellow/brown color with greater accuracy. Figure 9 shows the Total Ion Current (TIC) chromatogram, whereas Figure S4 of the Supplementary Material shows the mass spectra of the identified compounds. The MS spectra confirmed the NMR results, namely the presence of low molecular weight hydrocarbons as octadecane and hexadecane, and the presence of sulfur as sulfurous acid, and the identification of complex compounds, such as ethyl 5-chloro-2-nitrobenzoato and tributyl-chloro-stannane.

## 3. Discussion

Diagnostic analyses demonstrated that the black and white mosaic of the domus A was affected by a diffuse degradation of chemical origin, with the formation of gypsum crystals, due to the presence of sulfur in the topsoil beneath the mosaic bedding layer [18]. An aesthetic degradation was also evident because of organic compounds with 10–20 carbon atoms that could be traces of some unrefined combustive oil used in the past, but not yet present in the bedding layer, like the remains of up-to-date chemical pollutants used in pharmaceutical and agricultural industries [19].

These modern pollutants, as well as the sulfur traces, could have arisen from the aquifer beneath the mosaic room, permeating the overlying topsoil and consequently filtering through the mortar in the form of salts, or could be present in the soil bordering the mosaic room wall, which is still to be investigated. Another possible source of sulfur in the urban context could be sulfur oxide air pollution, in particular, due to wood heating. Still this system has by now almost completely disappeared in big cities such as Rome. Considering the wide micro-flora colonization of Palazzo Valentini underground settings, another hypothesis for the presence of sulfur could be special anoxygenic photosynthetic bacteria, such as Thiobacillus sp., able to oxidize sulfur (from sulfuric acid or other sulfur compounds produced during mineralization processes) to sulphates using the energy produced to fix organic carbon (CO_2_) in the photosynthetic process. Anaerobic sulfur bacteria can also partially oxidize sulfuric acid to elemental sulfur, which tends to form sediment in soils and takes part in long cyclic processes [20].

Based on the results of this study, three different hypotheses can be drawn on the reason for the deterioration. The first hypothesis is based on documents of the archaeological excavation where it is reported that, in the seventeenth century, some underground rooms of Palazzo Valentini, but at a higher level than the room of the *domus B* with the floor mosaic, were used as a printing house. Therefore, it can be hypothesized that the traces of hydrocarbons are attributable to printing oil percolating over time. A second hypothesis, on the other hand, is based on the presumed presence, reported in the chronicles of the seventeenth century, of a boiler in the room of the *domus B* and, therefore, on the fuel used for the same. The critical point of this hypothesis is that, although we have news of this boiler, it has never been found, much less traces of its possible presence. Finally, a third hypothesis on the origin of the degradation, perhaps more concrete and real, is based on more recent historical facts. Documentary research made during the excavation campaigns reports the use of these underground rooms as fuel oil storage for a boiler installed in proximity to the mosaic room. In addition, it is important to report that these subterranean settings were changed into a bunker during the Second World War, still existing as part of the exhibition itinerary, which extended to under *Palazzo delle Assicurazioni Generali di Venezia* and whose frequentation probably implied the use of some combustive substance for lighting and heating. This last hypothesis was confirmed in the finding, during the excavation, of metallic remains (lids, etc.) of drums of traction/heating fuel.

A possible definitive solution could be the isolation of mosaic pavement (or at a still unknown depth) from the underlying soil soaked with the organic compound. This kind of expensive intervention should involve the surviving mosaic’s detachment, the excavation of all back-up layers and an additional one to gain a tank far larger than the mosaic surface extension for the new waterproofing layer coating, without binder and of suitable thickness. The purpose of these steps would be reconstructing every bedding layer discovered during the detachment operation and re-installing the pre-existing mosaic, eventually replenishing it with tiles discovered in the new excavations (a similar approach has been chosen for the Giotto *frescoes* in the Upper Basilica of Assisi).

Finally, it would be really important to use compatible materials, as similar as possible to the original ones, such as ten-year aged hydrated lime, pozzolana, white marble powder, washed river sand and the like, recovering old recipes mentioned by Romans or during the Renaissance.

## 4. Materials and Methods

The sampling was performed at a few different points, in very small amounts, of course. After this, the samples were collected together and homogenized in a mortar and two representative samples were analyzed.

The experimental approach involved the use of multiple analytical methods for better identification of the compounds responsible for the degradation, namely Optical Microscope (OM), Scanning Electron Microscope with Energy Dispersion Spectroscopy (SEM-EDS), X-ray fluorescence (XRF), Nuclear Magnetic Resonance (NMR), Gas Chromatography coupled with Mass Spectroscopy (GC-MS) and Fourier Transform Infrared Spectroscopy (FT-IR). The authors opted for an analytical study to identify the substances involved and their common role in the degradation action. For this purpose, samples of the degradation product were taken from both the wall and the floor. Two samples of the degradation product were taken with tweezers and spatula, one from the floor and one from the wall (Figure 3a). The samples were observed under the Motic BA200 binocular optical microscope (Figure 3b) and in reflection mode with the Motic MLC-150C optical fiber. The instrument features were: 4×, 10×, 40×, and 100× quadruple nosepiece; focusing with a width of 25 mm, in 2 mm increments; 6 V/20 W halogen lighting system with light intensity control.

Samples were observed with SEM coupled with EDS to better investigate the crystal structure. The use of EDS made it possible to get a non-destructive qualitative and semi-quantitative sample analysis, identifying the chemical elements in the investigated spots unequivocally [21]. SEM analysis, over information on topography and crystal structure of the samples collected, also gave a grey-scale map of the surface, revealing zones with different medium atomic density (where the heaviest elements, which in our spectra was calcium, appeared clearer and brighter than those of lighter ones). As these were non-metallic samples, they needed to be coated with a conductive material during SEM sample preparation to make them compatible with SEM: a thin layer of gold was used by means of a sputter coater. Microscope analyses were performed by an SEM instrument (mod. Leica LEO 440 S, Wetzlar, Germany) equipped with an energy dispersive spectrometer for X-ray EDS (mod. INCA Energy 400, Oxford Instruments, Abingdon-on-Thames, UK). SEM allowed high magnification (beyond 100,000×) digital images of different kinds of samples with an up to 5-nm resolution while EDS allowed identification of the elemental composition, both of the entire sample and on more or less restricted areas. Our main experimental conditions were 10^−6^ hPa vacuum and 20 keV accelerating voltage. Diffractometry instead revealed the presence of crystalline compounds. This was carried out using a Bruker AXS D8 Focus automatic diffractometer for powders, operating in a reflection in Bragg Brentano θ/2θ geometry with an exposure of 12 h.

In addition, NMR analysis was performed over the degradation product samples from both pavement and wall of the mosaic room to analyze a possible organic component [22]. For NMR measurements, the samples taken from the pavimental mosaic and the wall were dissolved in deuterated chloroform (CDCl_3_), and after being centrifuged and filtered, they were subjected to ^1^H-NMR analysis. NMR analysis for the degradation product of the pavimental mosaic [23] was carried out using the mod. 300 MHz Varian Mercury (Varian, Palo Alto, CA, USA) operating at 300 MHz (superconducting magnet at 7.05 T). Chemical shifts in ^1^H-NMR spectra were reported in parts per million (ppm) on the δ scale using the solvent signal as an internal standard.

The Fourier transform infrared (FT-IR) spectroscopy was used to identify the organic compound structure by using characteristic vibrations of molecules producing spectra with characteristic bands in the infrared region (from 400 cm^−1^ to 4000 cm^−1^), where single bands can be attributed to vibrations of specific chemical groups. FT-IR spectra were carried out using an Alpha FT-IR instrument (Bruker Optics, Ettingen, Germany) equipped with a Globar source (i.e., a microscope) and a deuterated triglycine sulfate (DTGS) detector [24,25]. Each sample was analyzed, collecting 200 scans or more at a resolution of 2 cm^- 1^ in the spectroscopic range 4000–400 cm^−1^. The spectra collected showed excellent reproducibility [26].

Finally, a floor sample was analyzed by GC-MS, an analytic method by which complex mixtures of chemical compounds may be separated, and the compounds were identified and quantified. Each sample was dissolved in acetone and the analysis was performed by means of a HP-5890 Series II (HP, Rome, Italy) gas chromatograph coupled to a HP-5972 mass selective detector (HP). Chromatographic separations were achieved on a fused-silica capillary column (HP-5MS), stationary phase SE54 (5% phenyl-95% methylpolysiloxane), 30 m × 0.25 mm I.D. and 0.25 µm d*_f_* [27,28,29]. The chromatographic conditions were as follows: injector temperature 300 °C; injections made in splitless mode (30 s delay before opening the splitter); transfer line temperature 200 °C; initial oven temperature 120 °C, isothermal for 1 min; 30 °C min^−1^ up to 200 °C; 5 °C min^−1^ up 230 °C; then 30 °C min^−1^ up to the final temperature of 290 °C. The carrier gas was helium, constant inlet gas pressure of 5 psi. The mass spectrometer was scanned from *m/z* 50 to 700 at 70 eV.

## 5. Conclusions

This paper is a preliminary study on a black and white Roman mosaic. In particular, the authors wished to suggest a methodology for studying the state of degradation of an archaeological find. Such degradation, due to the presence of hydrocarbons, which to date cannot be dated with certainty, risk irreversibly damaging the mosaic, as well as the rooms and the walls in such *domus*. Different analytical methodologies were used to understand the organic composition involved in this contamination and the composition of the wall and pavement. The contamination, as well as the sulfur traces, could have arisen from the aquifer beneath the mosaic room, permeating the overlying topsoil and, consequently, filtering through the mortar in the form of salts, or could be present in the soil bordering the mosaic room wall, still not investigated. Another possible source of sulfur in the urban context could be sulfur oxide air pollution, in particular due to wood heating, but this system has by now almost completely disappeared in big cities like Rome. The authors believe that all these findings are fundamental for preliminary studies but they could also be very important for developing protocols of restoration and conservation. It should also be considered that during the writing of this paper, a cleaning intervention was made on the mosaic surface, but more invasive solutions should be applied in the event of a new occurrence of the above-mentioned degradation problems. A possibly definitive solution could be the isolation of mosaic pavement, or a still unknown depth, from the underlying soil soaked with the organic compound to ascertain it. This kind of expensive intervention should involve the surviving mosaic’s detachment, the excavation of all back-up layers and of an additional one to gain a tank far larger than the mosaic surface extension for a new waterproofing layer coating, without binder and of suitable thickness, with the purpose of then reconstructing every bedding layer discovered during thedetachment operation and re-installing the pre-existing mosaic, and eventually replenishing it with tiles discovered in the new excavations (a similar approach has been chosen for the Giotto frescoes in the Upper Basilica of Assisi).

## Figures and Tables

**Table 1:** EDS analysis (% *w/w*) of the wall and floor samples.

Spectrum	C	O	S	Ca	Mg	Al	Si	Total
Floor	16.5	47.9	14.6	21.0				100.0
Wall	25.9	51.1	4.9	16.7	0.3	0.3	0.8	100.0
A ^1^	24.7	47.6	11.1	16.6				100.0
B ^1^	25.9	56.1	7.8	9.8	0.4			100.0

^1^ Portions (particles “A” and “B”) of the wall sample as shown in Figure 4d.

## Data Availability

Not applicable.

## Supplementary Materials

The following supporting information can be downloaded at: https://www.mdpi.com/article/10.3390/molecules27227765/s1, Figure S1: “Veduta di Roma” (1593) by Antonio Tempesta: in the red box Palazzo Valentini is highlighted; Figure S2: Palazzo Valentini: (a) *domus A*: rests of the *peristilium* with black and white mosaic pavement and bases for columns or pilasters; (b) *domus A*: *triclinium* with colored geometric mosaic pavement; Figure S3. NMR spectra of the aliphatic hydrocarbons identified in the investigated samples; Figure S4. Selected Ion Monitoring (SIM) spectra of the compounds (octadecane, hexadecane, nonyl 2-propyl ester sulfurous acid, tributylchloro-stannane) investigated in the floor sample.

## Sample Availability

Samples are available from the authors.

## References

[1] Maltese C. (1991). Le Tecniche Artistiche.

[2] Baldassarri P., Del Signore R. (2008). Indagini archeologiche a Palazzo Valentini. La campagna 2005–2007. Palazzo Valentini. L’area tra Antichità ed età Moderna: Scoperte Archeologiche e Progetti di Valorizzazione.

[3] Quattrocchi M., Del Signore R. (2008). I mosaici della domus A. Palazzo Valentini. L’area tra Antichità ed età Moderna: Scoperte Archeologiche e Progetti di Valorizzazione.

[4] Baldassarri P., Şahin M. (2011). Archaeological excavations at Palazzo Valentini: A residential area in the shade of the Trajan’s Forum. Proceedings of the 11th International Colloquium on Ancient Mosaics.

[5] Acconci A., Baldassarri P., Nuzzo M., Tommasi F.M., Del Signore R. (2005). Profilo storico, artistico e archeologico di Palazzo Valentini. ‘La Provincia Capitale’. Storia di una Istituzione e dei suoi Presidenti.

[6] Cicconi M., Del Signore R. (2008). La ‘Fabbrica’ di Palazzo Bonelli-Valentini, residenza cardinalizia del Cinquecento. Il punto di partenza. Palazzo Valentini. L’area tra Antichità ed età Moderna: Scoperte Archeologiche e Progetti di Valorizzazione.

[7] Cola M.C. (2012). Palazzo Valentini in Roma: La committenza Zambeccari, Boncompagni, Bonelli tra Cinquecento e Settecento.

[8] Passigli S. (1989). Urbanizzazione e topografia di Roma nell’area dei Fori Imperiali tra XIV e XVI secolo. Mélanges L’école Française Rome.

[9] Google Earth. https://earth.google.com/web/@41.896258,12.48398616,35.16175745a,910.69330694d,35y,0h,0t,0r.

[10] Napoli L., Baldassarri P. (2015). Palazzo Valentini: Archaeological discoveries and redevelopment projects. Front. Archit. Res..

[11] Sammartino M.P., Visco G. (2014). Led Lighting Installation and Related Process for Inhibiting the Development of Photosynthetic Biodeteriogen Organisms in Hypogeal Environments.

[12] Sudmalis M., Sheikholeslami R. (2000). Precipitation and co-precipitation of CaCO_3_ and CaSO_4_. Can. J. Chem. Eng..

[13] Faulstich F.R.L., Schnellrath J., De Oliveira L.F., Scholz R. (2013). Rockbridgeite inclusion in rock crystal from Galileia region, Minas Gerais, Brazil. Eur. J. Mineral..

[14] Sudoł E., Małek M., Jackowski M., Czarnecki M., Strąk C. (2021). What makes a floor slippery? A brief experimental study of ceramic tiles slip resistance depending on their properties and surface conditions. Materials.

[15] Mason J. (1981). Nitrogen nuclear magnetic resonance spectroscopy in inorganic, organometallic, and bioinorganic chemistry. Chem. Rev..

[16] Fulmer G.R., Miller A.J.M., Sherden N.H., Gottlieb H.E., Nudelman A., Stoltz B.M., Bercaw J.E., Goldberg K.I. (2010). NMR chemical shifts of trace impurities: Common laboratory solvents, organics, and gases in deuterated solvents relevant to the organometallic chemist. Organometallics.

[17] Babij N.R., McCusker E.O., Whiteker G.T., Canturk B., Choy N., Creemer L.C., De Amicis C.V., Hewlett N.M., Johnson P.L., Knobelsdorf J.A. (2016). NMR chemical shifts of trace impurities: Industrially preferred solvents used in process and green chemistry. Org. Process Res. Dev..

[18] Van Driessche A.E.S., Stawski T., Kellermeier M. (2019). Calcium sulfate precipitation pathways in natural and engineering environments. Chem. Geol..

[19] Stepanova A.Y., Gladkov E.A., Osipova E.S., Gladkova O.V., Tereshonok D.V. (2022). Bioremediation of Soil from Petroleum Contamination. Processes.

[20] Waluś K.L., Warguła L., Wieczorek B., Krawiec P. (2022). Slip risk analysis on the surface of floors in public utility buildings. J. Build. Eng..

[21] Nasrazadani S., Hassani S., Makhlouf A.S.H., Aliofkhazraei M. (2016). Modern analytical techniques in failure analysis of aerospace, chemical, and oil and gas industries. Handbook of Materials Failure Analysis with Case Studies from the Oil and Gas Industry.

[22] Bifulco G., Dambruoso P., Gomez-Paloma L., Riccio R. (2007). Determination of relative configuration in organic compounds by NMR spectroscopy and computational methods. Chem. Rev..

[23] Lazzari M., Reggio D. (2021). What fate for plastics in artworks? An overview of their identification and degradative behavior. Polymers.

[24] Tarquini G., Nunziante-Cesaro S., Campanella L. (2014). Identification of oil residues in Roman amphorae (Monte Testaccio, Rome): A comparative FTIR spectroscopic study of archeological and artificially aged samples. Talanta.

[25] Nunziante-Cesaro S., Lemorini C. (2012). The function of prehistoric lithic tools: A combined study of use-wear analysis and FTIR microspectroscopy. Spectrochim. Acta A.

[26] Nucara A., Nunziante-Cesaro S., Venditti F., Lemorini C. (2020). A multivariate analysis for enhancing the interpretation of infrared spectra of plant residues on lithic artefacts. J. Archaeol. Sci. Rep..

[27] Russo M.V., Avino P., Cinelli G., Notardonato I. (2012). Sampling of organophosphorus pesticides at trace levels in the atmosphere using XAD-2 adsorbent and analysis by gas chromatography coupled with nitrogen-phosphorus and ion-trap mass spectrometry detectors. Anal. Bioanal. Chem..

[28] Russo M.V., Notardonato I., Avino P., Cinelli G. (2014). Fast determination of phthalate ester residues in soft drinks and light alcoholic beverages by ultrasound/vortex assisted dispersive liquid-liquid microextraction followed by gas chromatography-ion trap mass spectrometry. RSC Adv..

[29] Avino P., Notardonato I., Perugini L., Russo M.V. (2017). New protocol based on high-volume sampling followed by DLLME-GC-IT/MS for determining PAHs at ultra-trace levels in surface water samples. Microchem. J..

