# Supplementary material for: Visual and Physical Degradation of the Black and White Mosaic of a Roman Domus under Palazzo Valentini in Rome: A Preliminary Study
Source: Molecules. 2022 Nov 11;27(22):7765. doi: 10.3390/molecules27227765 (PMC9699458; doi:10.3390/molecules27227765)
Supplement: Supplementary file 1 [file molecules-27-07765-s001.zip › molecules-1970547-supplementary.pdf]

Supplementary Material

Visual and Physical Degradation of the Black and White Mosaic of a *Roman Domus* under Palazzo Valentini in Rome: A Preliminary Study

Claudia Colantonio [1], Paola Baldassarri [2], Pasquale Avino [3,*], Maria Luisa Astolfi [1], Giovanni Visco[1]

[1] Department of Chemistry, University of Rome "La Sapienza", p.le Aldo Moro 5, I-00185 Rome, Italy

[2] Città Metropolitana di Roma Capitale, U.C. 2, Servizio 3, Palazzo Valentini, Via Quattro Novembre 119a, I-00187 Rome, Italy

[3] Department of Agricultural, Environmental and Food Sciences (DiAAA), University of Molise, Via De Sanctis, I-86100 Campobasso, Italy

[4] CIABC, University of Rome "La Sapienza", p.le Aldo Moro 5, I-00185 Rome, Italy

[*] Correspondence: avino@unimol.it

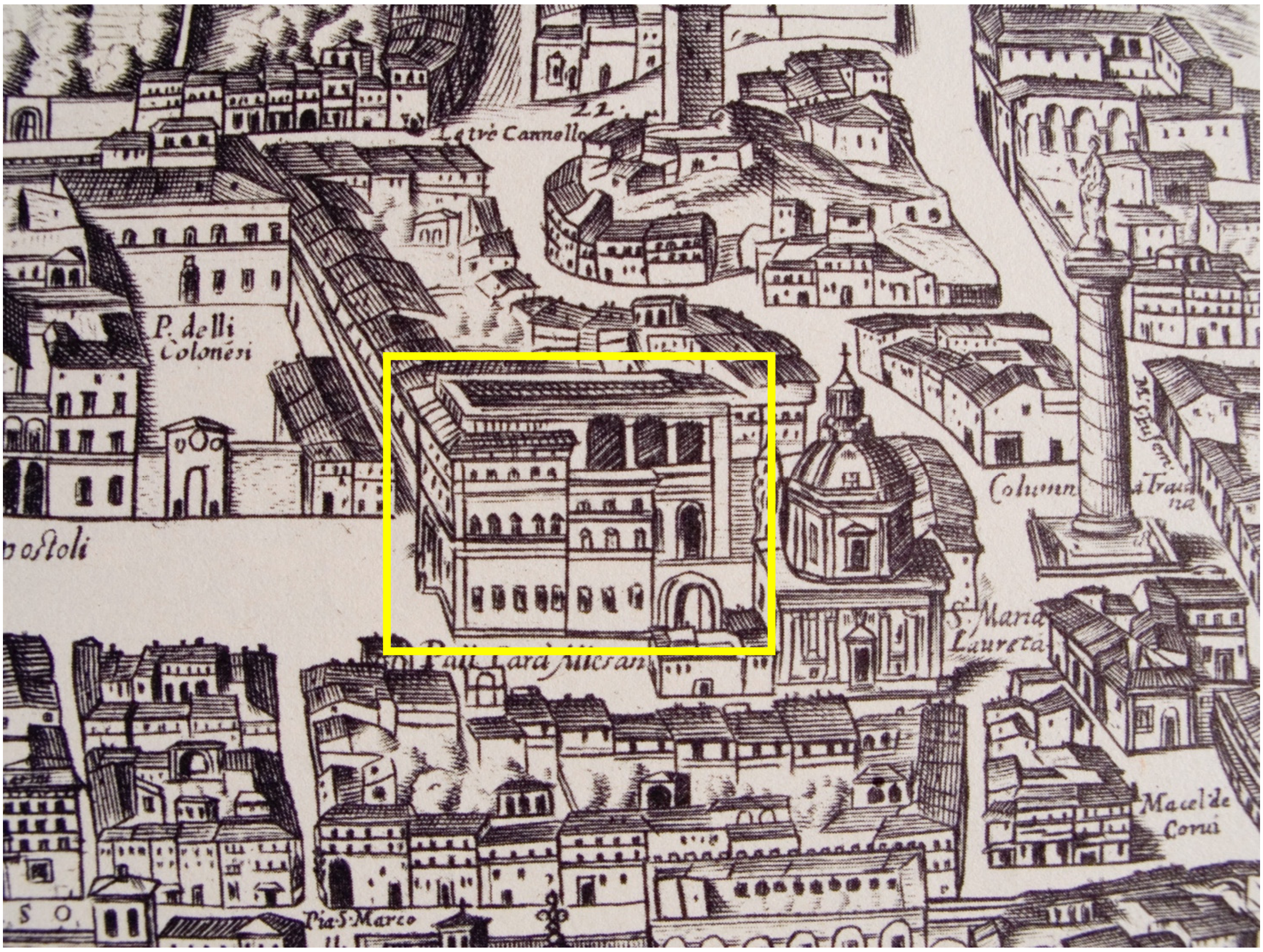

Figure S1. “Veduta di Roma” (1593) by Antonio Tempesta: in the yellow box Palazzo Valentini is highlighted.

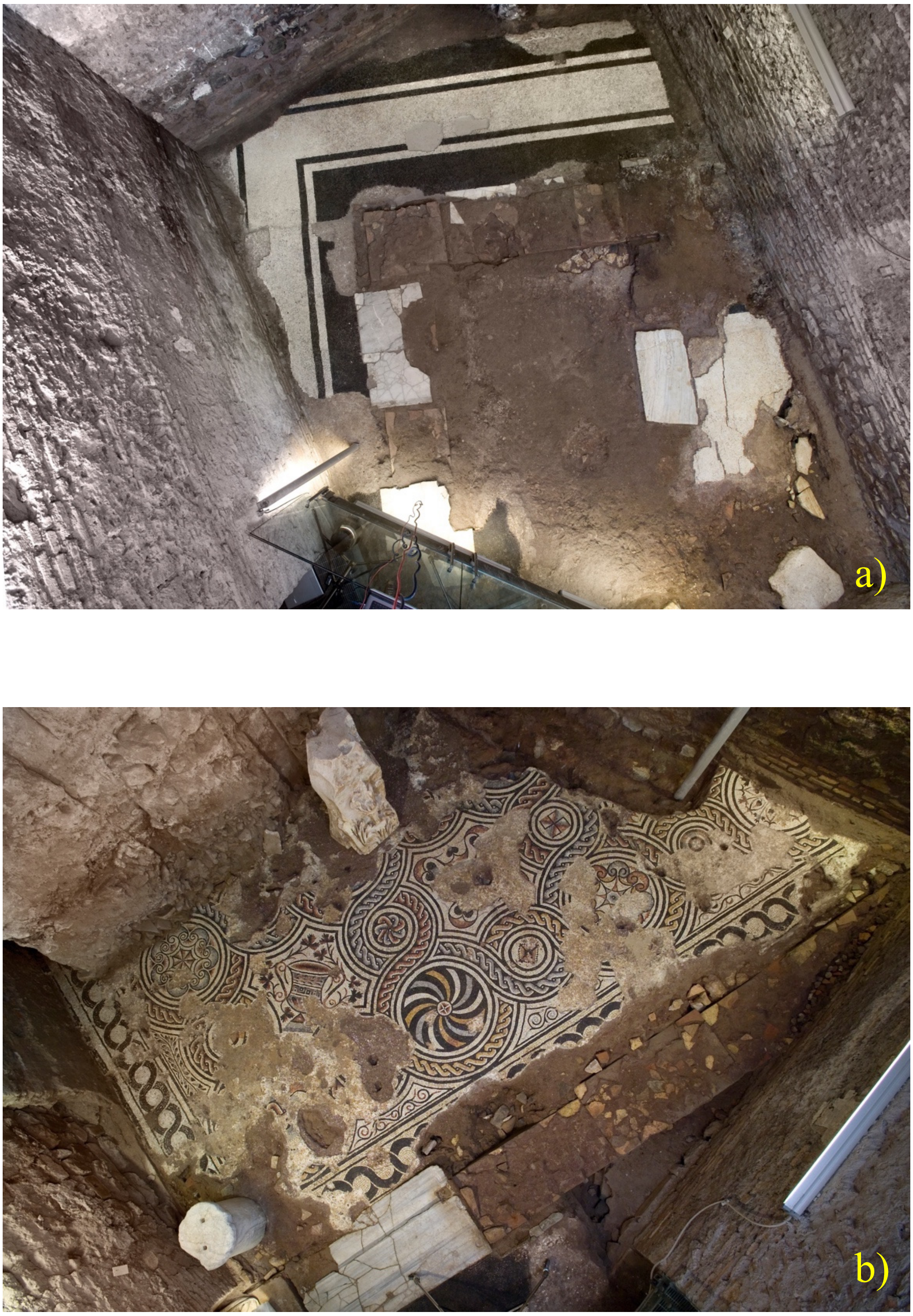

Figure S2. Palazzo Valentini: a) *domus A*: rests of the *peristilium* with black and white mosaic pavement and bases for columns or pilasters; b) *domus A*: *triclinium* with colored geometric mosaic pavement.

SDBS-^{1}H NMRSDBS No. 4461HSP-03-643

89.56 MHz

$C_8 H_{19} N$

0.04 ml : 0.5 ml $CDCl_3$

1-methylheptylamine

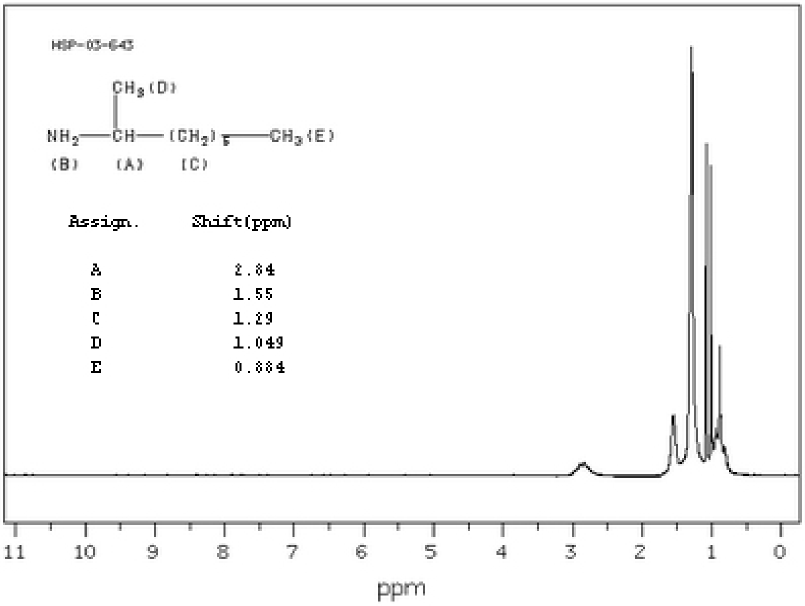

SDBS-^{1}H NMRSDBS No. 6055HSP-03-715

89.56 MHz

$C_7 H_{16}$

0.04 ml : 0.5 ml $CDCl_3$

2-methylhexane

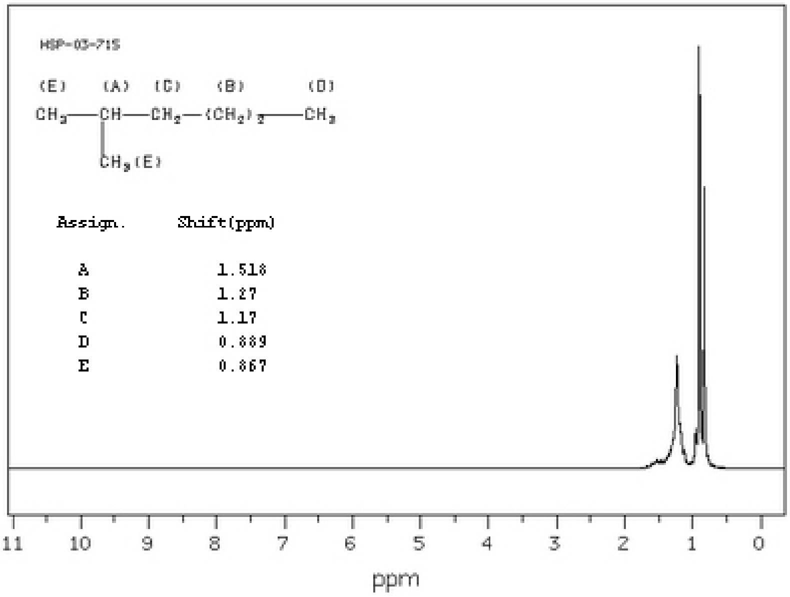

SDBS-¹H NMR SDBS No. 197HSP-01-976

89.56 MHz

$C_{10}H_{22}$

0.04 ml : 0.5 ml $CDCl_3$

2-methylnonane

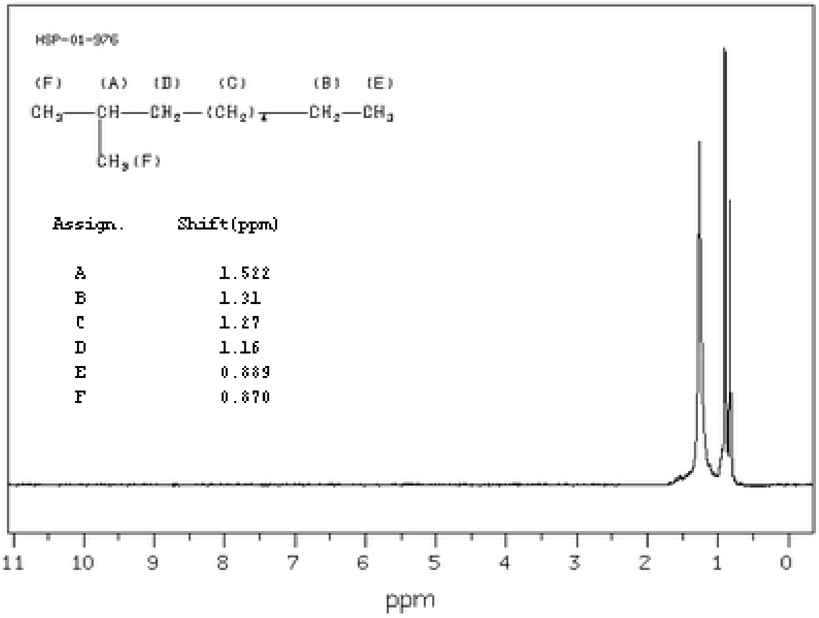

SDBS-¹H NMR SDBS No. 6866HSP-01-789

89.56 MHz

$C_{10}H_{23}N$

0.04 ml : 0.5 ml $CDCl_3$

decylamine

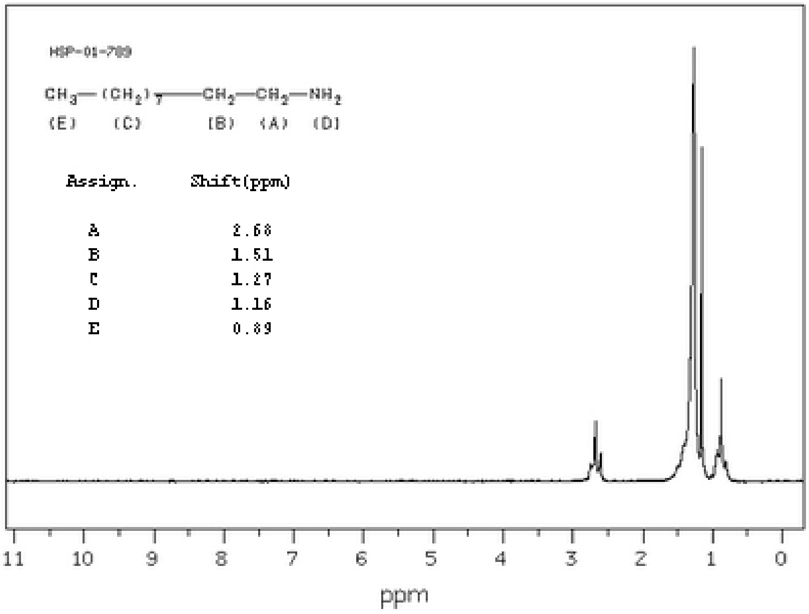

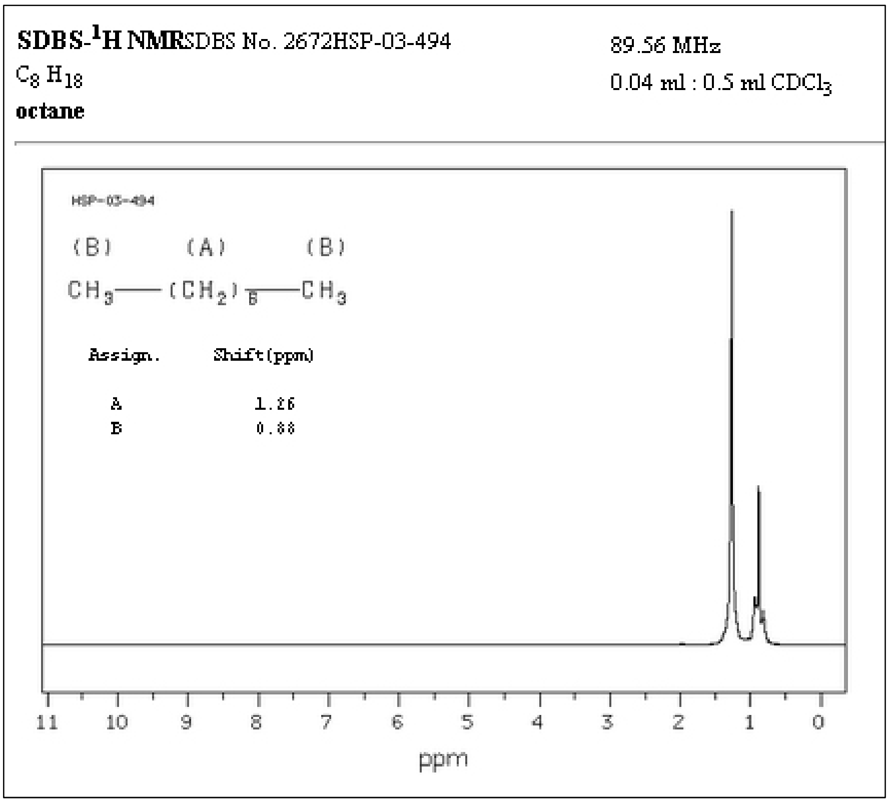

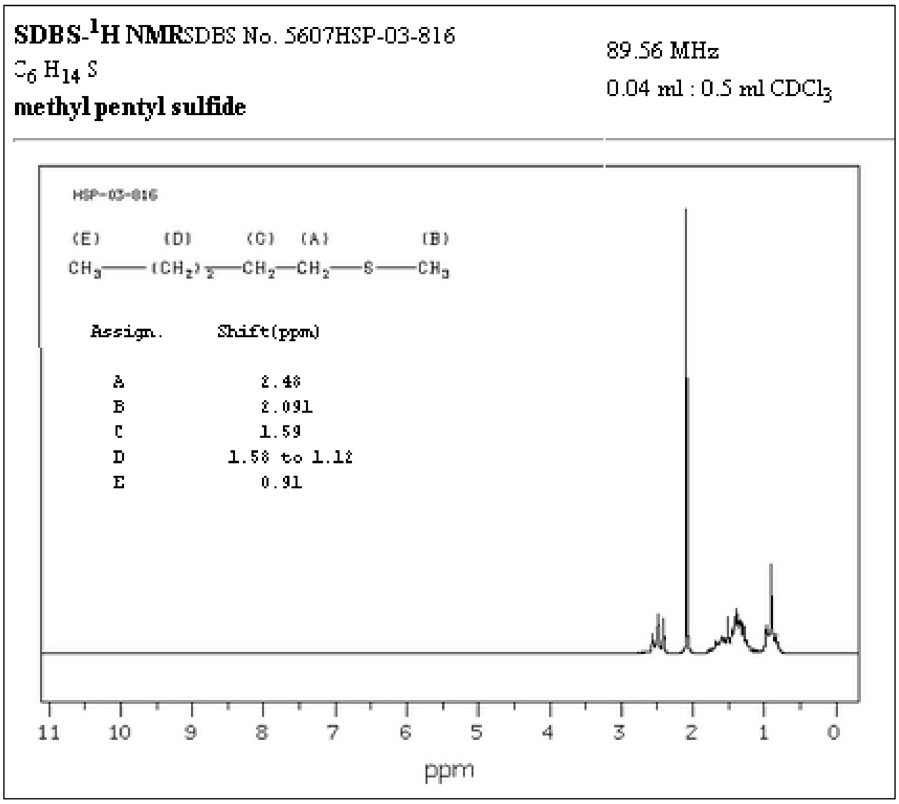

Figure S3. Spectra of the aliphatic hydrocarbons identified in the investigated samples (source: SDBS).

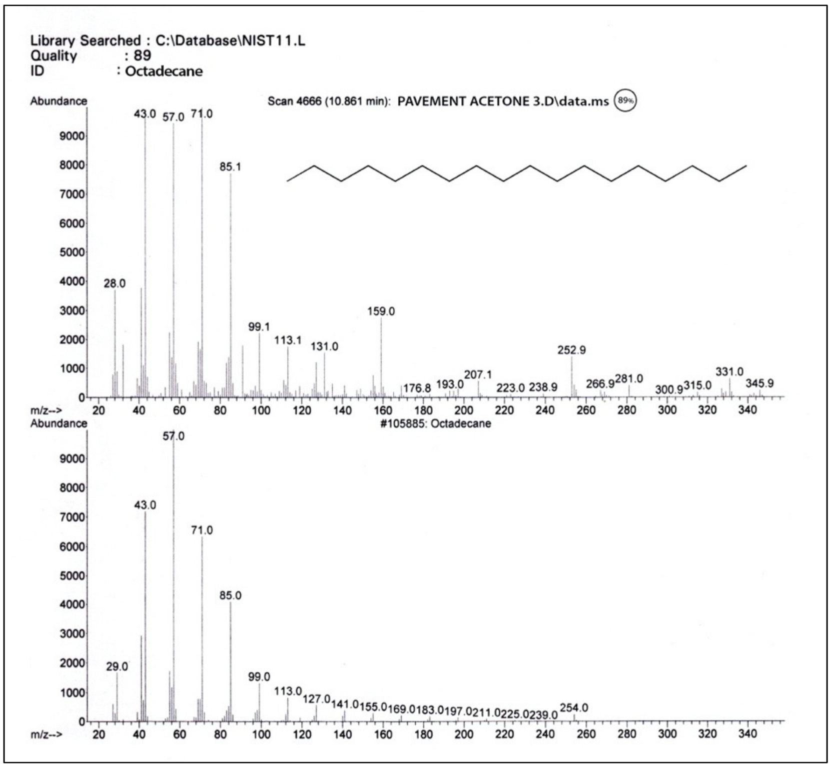
Library Searched : C:\Database\NIST11.L
Quality : 89
ID : Octadecane
Abundance
Scan 4666 (10.861 min): PAVEMENT ACETONE 3.D\data.ms 89%
m/z-->
#105885: Octadecane

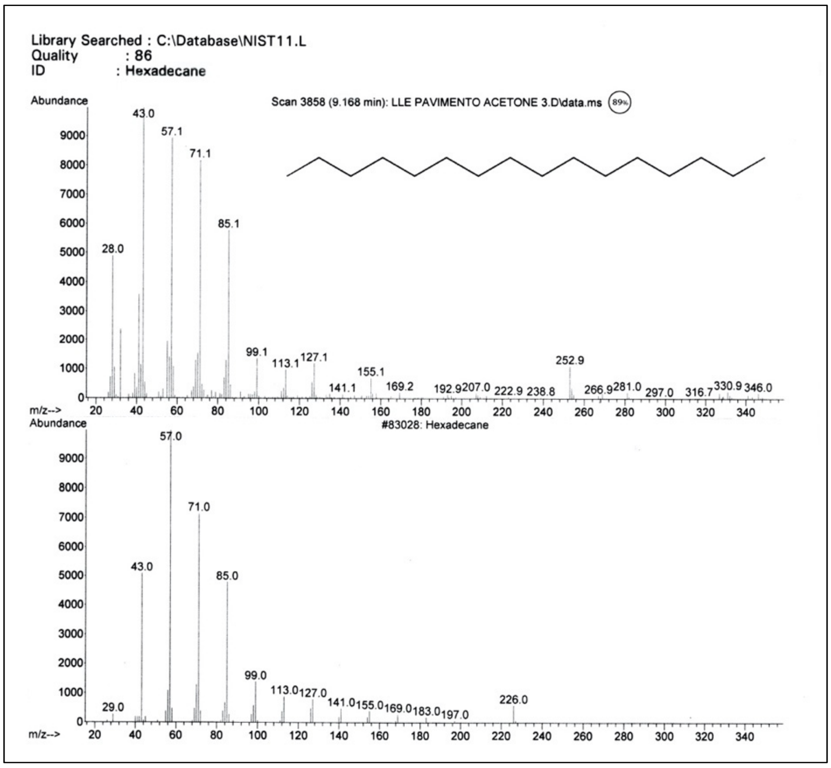
Library Searched : C:\Database\NIST11.L
Quality : 86
ID : Hexadecane
Abundance
Scan 3858 (9.168 min): LLE PAVIMENTO ACETONE 3.D\data.ms 89%
m/z-->
#83028: Hexadecane

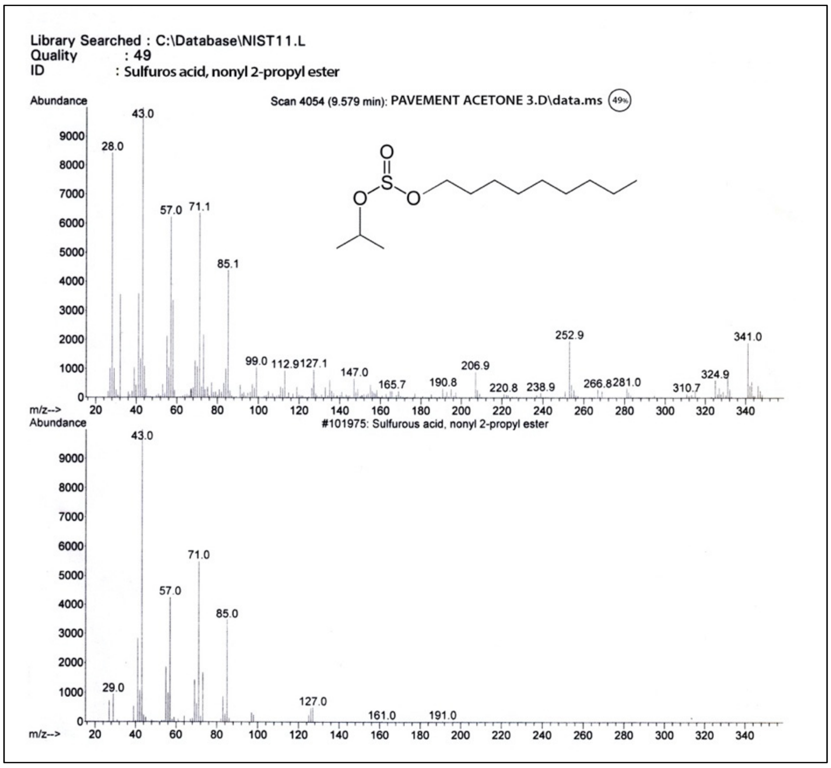

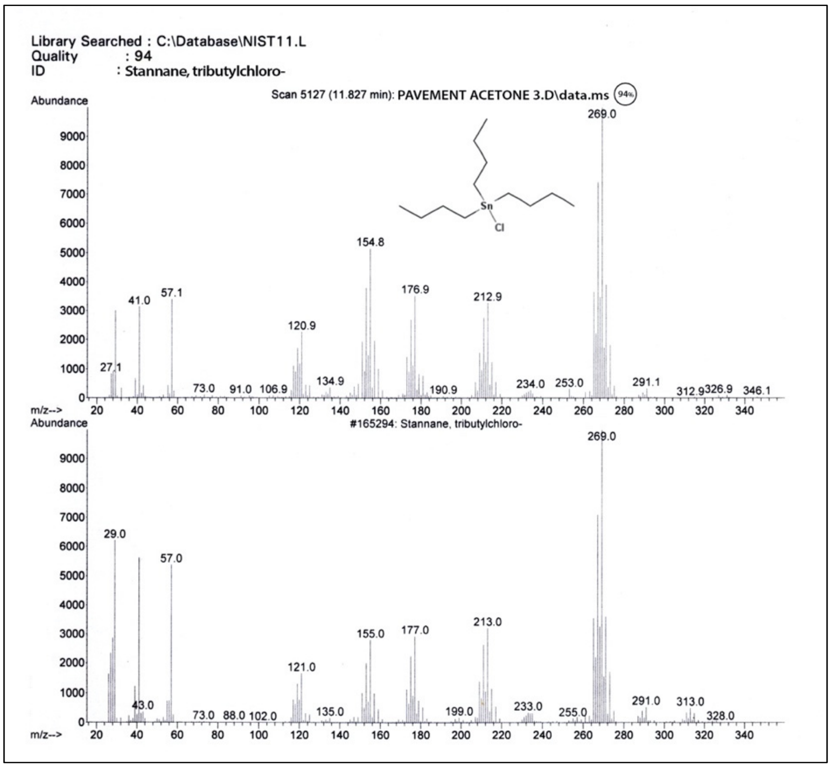

Figure S4. Massa spectra of the compounds (octadecane, hexadecane, nonyl 2-propyl ester sulfurous acid, tributylchloro-stannane) investigated in the floor sample.
